# Does a placebo effect really occur in dogs afflicted by hip osteoarthritis as measured by force platform gait analysis?

**DOI:** 10.1186/1746-6148-9-260

**Published:** 2013-12-21

**Authors:** Maxim Moreau, Bertrand Lussier, Jean-Pierre Pelletier, Eric Troncy

**Affiliations:** 1 Osteoarthritis Research Unit, Université de Montréal Hospital Centre, Tour Viger, 900 rue St-Denis, Montreal, Quebec H2X 0A9, Canada; 2 GREPAQ, Department of Veterinary Biomedical Sciences – Faculty of Veterinary Medicine, Université de Montréal, P.O. Box 5000, Saint-Hyacinthe, Quebec J2S 7C6, Canada; 3 Department of Clinical Sciences –Faculty of Veterinary Medicine, Université de Montréal, P.O. Box 5000, Saint-Hyacinthe, Quebec J2S 7C6, Canada

**Keywords:** Effect size, Dog, Osteoarthritis, Placebo effect, Peak vertical force

## Abstract

A recent study investigated the therapeutic response of dogs afflicted by hip osteoarthritis when evaluating therapeutic modalities compared to a negative (placebo) control group. Authors suggested a placebo effect based on peak vertical force measurement. In addition, small effect size for each of the tested therapeutics as well as the extremely large sample size needed (>450) to discern therapeutic efficacy using force platform gait analysis were reported. We wish to express our concerns regarding the eligibility criteria used to select the studied cohort, the small effect size, and the placebo effect reported in force platform gait analysis.

## 

We would like to provide some comments on the recently published article on the clinical outcome measures in a randomized controlled trial (RCT) of canine osteoarthritis (OA) from the perspective of clinical investigators. The article investigated the therapeutic response of dogs afflicted by hip OA to three therapeutic modalities compared to a negative (placebo) control group [1]. The main goal of the study was to determine the effect size (ES) of key outcome measures and, secondly, to highlight, in such RCTs assessing different treatments, the interest of combining multiple (of different nature) outcome measures. Effect size emphasises the size of the difference rather than confounding this with sample size as in statistical significance. However, it is very rarely used in primary reports. Due to the unfamiliarity of using this test in such a context, we would like to call for caution before a conclusion can be drawn from this study.

As indicated in their Introduction, in a recent meta-analysis of RCTs in human OA, Zhang *et al*. [2] reported a placebo ES (the standard mean difference between baseline and endpoint) of 0.51 (95% CI 0.46 to 0.55). Effect sizes can be interpreted in terms of the percentiles or ranks at which two distributions overlap [3]. With an ES of 0.51, the probability that one could guess which group (naïve at baseline, or placebo at endpoint) a person was in based on their ‘score’ is around 60%, whereas an ES of 0.00 would logically provide a similar probability of 50%. This is, at least in our judgement, a more relative interpretation of a ‘mild’ ES (0.20 < ES < 0.80); a placebo ES of 0.51 gives only a 10% increased chance of determining the group of the examined person. Two other points need to be reported from the study of Zhang *et al*. [2]. They found placebo to be effective in all subjective outcomes (not just patient-associated [*e.g*. pain, stiffness, self-reported function] but also observer-centred [doctor global opinion]), but ineffective for almost all objective outcomes (*e.g*. quadriceps strength, knee circumference, range of movement, radiographic narrowing); the ES of placebo was twice as high in hand OA (0.80) compared to hip OA (0.37).

To reach their goal, Malek *et al. *[1] recruited dogs having hip OA in addition to concomitant musculoskeletal conditions (n = 27/49, 55% of included dogs). Several dogs (n = 7) had surgically altered hip or stifle joint structures including excision arthroplasty and total hip replacement. We believe that inclusion criteria should have been more restrictive to homogenise the studied cohort and to avoid any distinct gait pattern or joint biomechanical changes, thus preserving statistical power and maximising ES. To avoid therapeutic plateau or positive bias in outcome measures, which could be particularly harmful to the ES, we also believe in the necessity of carefully defined wash-out periods for OA therapeutics including joint supplements or diets (n = 24/49) purported to improve afflicted dogs. Several peer-reviewed studies published in the last few years have respected the implementation of wash-out periods for sporadic non-steroidal anti-inflammatory drugs (NSAIDS), oral nutraceuticals, OA therapeutic diets, fatty acid supplements, continuous oral or injectable anti-inflammatory drugs (including both steroids and NSAIDs), or polysulfated glycosaminoglycan therapy [4-13].

Regarding the force platform gait analysis, the limb with the smallest F_z_ vector (later referred to as peak vertical force by Malek *et al. *[1]) was analysed, which does not necessarily mean that this parameter departed from normality. This was particularly critical considering the large heterogeneity in the cohort of recruited dogs. One would suggest, rather, a selection of subjects based on a predetermined variable threshold [4,6,9,14]. This threshold can be determined *a priori* with respect to normal values [13]. We would also like to suggest a selection of trials according to a velocity range limited to 0.3 m/s (*e.g*. 1.6-1.9 or 1.9-2.2 m/s at the trot) [4,6-12,14] instead of a larger one that overlaps walking and trotting gait intervals (*e.g*. 1.3-1.9 m/s). For the measurement of pelvic gait parameters, conditions that predispose dogs to thoracic limb pain or functional abnormalities, such as elbow and shoulder OA, should be discarded to limit force redistribution to the pelvic limbs [11]. Furthermore, no documentation of the body weight was recorded at the end of the study. It is believed that this point is of importance as changes in individual body weight can affect outcome measures in OA dogs [15].

Is it unclear why Malek *et al. *[1] interpreted the changes in falling slope observed in placebo-treated dogs as being “an undesired effect” and “due to pain” (see Malek *et al. *[1], Tables eight and eleven) when the authors later concluded a placebo effect in dogs with hip OA after having denoted a mean (standard deviation, SD) change in peak vertical measurement of 2.8 (10.6)% body weight (see Malek *et al. *[1], Table eight). Without precise information on the stance phase duration, it is difficult to fully integrate falling slope changes. As gait parameters are intimately linked, it is strongly suggested to make *a priori* determination of the primary outcome to avoid any misleading interpretation.

We would like to express our disagreement with a sole representation of force platform gait analysis data as change in percentage relative to the initial condition. Rather, we suggest group central tendencies at each end-point as well as individual changes. Such representation could have been useful to demonstrate the regression to the mean phenomenon hypothesised by the authors [1] in an attempt to support a placebo effect in dogs with hip OA upon force platform gait analysis. Moreover, we believe that the accumulation of previously listed methodological short cuts could explain the large variability in kinetic (force platform) parameters observed in the study [1]. The variability of the results is reflected by the large SD values in kinetic parameters reported in Malek *et al. *[1], Table eight.

As mentioned by Malek *et al. *[1], dogs showing a positive response to a negative control (placebo) were previously observed based on peak vertical [6,16], but globally the ground reaction forces did not demonstrate clinically meaningful changes under placebo and rather tend to be slightly negative [9,10,12,13]. This was confirmed by a recent meta-analysis including 40 OA dogs followed over 28 days [mean (SD) change -1.5 (3.1)% body weight with an intra-class coefficient of correlation of 91, and only 5 dogs presenting a real positive response for peak vertical force] [17] and a recent prospective study including 58 OA dogs over 42 days [18]. These latter studies reported a rate of positive responses around 10% using peak vertical force measurement.

Moreover, our concern about the negative interference of the methodology used by Malek *et al*. [1] with the kinetic measurement is supported by the absence of response to carprofen, which could be considered a positive control. Three previous publications reported positive response on peak vertical force in n = 36, n = 15 and n = 16, respectively, OA dogs treated with carprofen for 14 [16], 56 [13] and 60 [11] days. These findings could be related to a high proportion of neuropathic dogs in the recruited sample as mentioned by Malek *et al. *[1]. Hence, neuropathic pain is recognized as non-responsive to NSAIDS [19] and a component of a chronic painful condition such as osteoarthritis in dogs [20,21]. It is unfortunate that hyperalgesia/allodynia was not specifically tested in this population of dogs with the use, for instance, of von Frey anesthesiometer-induced paw withdrawal threshold, as recently reported in OA cats [22].

We are in agreement with the clinical relevance of reporting ES and the paramount importance of statistical power and sample size estimates to design fruitful RCTs. However, we encourage a thorough presentation of the method used for single group ES calculation [23] from which Malek *et al. *[1] derived their conclusions. The interest of this publication remains in the original report of a TRPV1 antagonist (ABT-116) and tramadol efficacy in canine OA. However, the poor ES reported for each of the tested therapeutics as well as the extremely large sample size needed (>450) to discern efficacy of force platform gait analysis, should be considered with scepticism. Furthermore, the data did not support placebo (or nocebo) effects in dogs with hip OA upon force platform gait analysis.

Response

By Peter Muir

E-mail: muirp@vetmed.wisc.edu

Address: Comparative Orthopaedic Research Laboratory, University of Wisconsin-Madison, School of Veterinary Medicine, 2015 Linden Drive, Madison, WI 53706, USA. Force-plate analysis-of-gait is one of several different outcome measures used in clinical trials studying client-owned dogs with osteoarthritis. Approaches to experimental design and statistical analysis of data in force-plate analysis-of-gait studies vary widely in the veterinary literature and often vary from the perspective of Moreau *et al*. For example, there are at least 10 different published velocity ranges used for force-plate analysis-of-gait in dogs, including a recent paper published in the *Journal of Veterinary Medical Association* that used a velocity range of 1.3-2.1 m/s [24].

The main objective of our study was to develop a screening assay in client-owned dogs for analgesic activity of new compounds for the treatment of human and veterinary osteoarthritis. As such, the number of animals and the duration of the study were kept to a minimum. Other studies have often used larger group sizes and longer durations of treatment. In our study, effect size determinations were used to establish which of the measured variables could best detect differences among treatment groups. In undertaking a treatment experiment, trial design may be restrictive or model-based, such that a test article may show a treatment effect that is subsequently less evident in clinical practice. Trials that more closely model clinical practice represent a greater treatment challenge for a test article to overcome. Whilst homogenous cohorts are often used in experimental osteoarthritis studies, client-owned dogs represent a more heterogeneous, but more clinically relevant, population for longitudinal veterinary treatment studies. For example, lumbosacral abnormalities in dogs are very common and may not always have been identified in past osteoarthritis studies in client-owned dogs. Lumbosacral spondylosis is often an incidental finding in clinically normal dogs, but may also be associated with clinical signs. Neuropathic pain as a clinical syndrome is poorly recognized in dogs [19] and its association with lumbosacral spondylosis is unclear. The responsiveness of dogs in the present study to carprofen, as measured by the Canine Brief Pain Inventory questionnaire [25], suggests that neuropathic pain was not an important phenomenon in our study.

Our study highlights the value of examining a combination of clinical outcome measures in clinically relevant longitudinal studies of client-owned dogs with osteoarthritis. Our study also highlights the need for better analgesic treatments for canine osteoarthritis in veterinary medicine. In this short-term study, habitual diet was maintained to minimize change in body weight over time. Whilst improvement in some gait analysis parameters was identified with placebo treatment in our study, these effects were not statistically significant. Some individual dogs showed an improvement in measured parameters with carprofen treatment, similar to a previous report, in which 19 of 34 dogs showed improvement in ground reaction forces with placebo treatment [16].

To summarize, the article by Moreau *et al*. contributes to discussion of force-plate analysis-of-gait as an outcome measure in longitudinal studies in client-owned dogs with osteoarthritis. Their commentary does not affect our conclusions regarding its importance in small screening studies.

## Abbreviations

RCT: Randomized controlled trial; OA: Osteoarthritis; ES: Effect size; NSAIDS: Non-steroidal anti-inflammatory drugs; SD: Standard deviation.

## Competing interests

The authors declare that they have no competing interests.

## Authors’ contributions

MM drafted the manuscript. BL, JPP and ET reviewed, commented and complemented the manuscript. All authors have read and approved the final manuscript.

## Authors’ information

MM is experienced in force platform gait analysis in canine and feline naturally-occurring and canine experimentally induced osteoarthritis.

BL, JPP and ET are full professors at the Université de Montréal, and present several decades of expertise in canine and feline osteoarthritis.

JPP and ET are heads of the Osteoarthritis Research Unit and GREPAQ, respectively.
